# Glucose Tolerance,* MTHFR* C677T and* NOS3* G894T Polymorphisms, and Global DNA Methylation in Mixed Ancestry African Individuals

**DOI:** 10.1155/2016/8738072

**Published:** 2016-11-20

**Authors:** Tandi E. Matsha, Carmen Pheiffer, Tinashe Mutize, Rajiv T. Erasmus, Andre P. Kengne

**Affiliations:** ^1^Department of Biomedical Sciences, Faculty of Health and Wellness Sciences, Cape Peninsula University of Technology, Cape Town, South Africa; ^2^Biomedical Research and Innovation Platform, South African Medical Research Council, Cape Town, South Africa; ^3^Division of Chemical Pathology, Faculty of Medicine and Health Sciences, National Health Laboratory Service (NHLS) and University of Stellenbosch, Cape Town, South Africa; ^4^Non-Communicable Diseases Research Unit, South African Medical Research Council, Cape Town, South Africa; ^5^Department of Medicine, University of Cape Town, Cape Town, South Africa

## Abstract

The aim of this study is to quantify global DNA methylation and investigate the relationship with diabetes status and polymorphisms in MTHFR C677T and NOS3 G894T genes in mixed ancestry subjects from South Africa. Global DNA methylation was measured, and* MTHFR* rs1801133 and* NOS3* rs1799983 polymorphisms were genotyped using high throughput real-time polymerase chain reaction and direct DNA sequencing. Of the 564 participants, 158 (28%) individuals had T2DM of which 97 (17.2%) were screen-detected cases. Another 119 (21.1%) had prediabetes, that is, impaired fasting glucose, impaired glucose tolerance, or the combination of both, and the remainder 287 (50.9%) had normal glucose tolerance. Global DNA methylation was significantly higher in prediabetes and screen-detected diabetes than in normal glucose tolerance (both *p* ≤ 0.033) and in screen-detected diabetes compared to known diabetes on treatment (*p* = 0.019). There was no difference in global DNA methylation between known diabetes on treatment and normal glucose tolerance (*p* > 0.999). In multivariable linear regression analysis, only* NOS3* was associated with increasing global DNA methylation (*β* = 0.943; 95% CI: 0.286 to 1.560). The association of global DNA methylation with screen-detected diabetes but not treated diabetes suggests that glucose control agents to some extent may be reversing DNA methylation. The association between* NOS3* rs1799983 polymorphisms and DNA methylation suggests gene-epigenetic mechanisms through which vascular diabetes complications develop despite adequate metabolic control.

## 1. Introduction

Type 2 diabetes mellitus (T2DM) results from an interaction of environmental and genetic factors. Despite the initial enthusiasm from the identification of risk loci for T2DM [1, 2], the clinical utility of these genetic markers for T2DM risk prediction and reduction has remained limited. It is becoming increasingly evident that the gene-environment interaction in T2DM and other diseases can be explained in part by epigenetics. Indeed, a number of recent studies using different types of biological tissues from pancreas to peripheral blood mononuclear cells (PBMCs) are showing that DNA methylation patterns are altered in subjects with diabetes [3–6]. T2DM rates are increasing rapidly in African populations, with traditional diabetes risk factors failing to explain a great deal of these increases. Emerging alternate pathways involve the interplay between epigenetics, microbiome, and the immune system. Epigenetic mechanisms include DNA methylation, lysine methylation, histone methylation, histone phosphorylation, RNA interference (RNAi), and genomic imprinting [7]. DNA methylation is the most extensively investigated epigenetic mechanism; however, DNA methylation profiling of populations from Africa remains to be investigated.

DNA methylation is an epigenetic process characterized by a covalent modification with the addition of a methyl group to the carbon at position 5 of cytosine nucleotides (H5), process catalyzed DNA methyltransferases (DNMTs) [8]. S-Adenosyl-methionine (SAM-CH3) donates the methyl group in a process catalyzed by methylenetetrahydrofolate reductase (MTHFR), an important enzyme in the folate metabolic pathway. Collectively, this folate and methionine cycles form the transmethylation pathway, which is modulated by a number of genetic and environmental factors that in turn affect DNA methylation [9]. In this regard, DNA hypomethylation has been reported in liver biopsies of subjects with type 2 diabetes (T2DM) with low folate levels [10], while* MTHFR* polymorphisms have been associated with decreased enzyme activity. Two* MTHFR* polymorphisms, +677C/T (rs1801133) and +1298A/C (rs1801131), have been widely studied and in vitro studies have shown these polymorphisms to decrease the enzyme activity by 35% and 60%, respectively [11, 12], consequently causing DNA hypomethylation. Another gene that has been shown to be affected by folate supplementation is nitric oxide synthase (NOS) [13, 14]. Nitric oxide synthase (eNOS, also referred to as NOS3) is an endothelial enzyme whose function is to synthesize nitric oxide (NO) from L-arginine [15, 16]. Nitric oxide is a unique molecule with diverse physiologic regulatory functions such as smooth muscle relaxation, inhibition of platelet aggregation, immune regulation, neurotransmission, and blood pressure regulation [17–19].

Therefore, this study was designed to examine global DNA methylation in individuals with and without diabetes and to evaluate whether polymorphisms in genes involved in DNA methylation and/or folate metabolism, namely,* MTHFR and NOS3*, have an effect on global DNA methylation in mixed ancestry subjects from South Africa.

## 2. Materials and Methods

### 2.1. Ethical Approval of the Study

The study was approved by the Faculty of Health and Wellness Sciences Ethics Committee of the Cape Peninsula University of Technology (CPUT) (NHREC: REC-230408-014) and was conducted according to the code of ethics of the World Medical Association (Declaration of Helsinki, 1975). All participants who were recruited for the study voluntarily signed written consent after the procedures had been fully explained in the language of their choice. Permission to conduct the study was granted by relevant authorities such as city and community authorities as previously described [20].

### 2.2. Study Design and Population

The present study was cross-sectional by design, involving participants from a mixed ancestry ethnic population group residing in Bellville South Township in Cape Town, South Africa. A detailed description of the survey and procedures conducted in the study are available elsewhere [21, 22]. Eligible participants were those who are older than 20 years, are residing in Bellville South, are of mixed ancestry origin, are not pregnant, and are not acutely ill.

### 2.3. Clinical Data

Clinical data which has been previously described [20] was collected in the form of a standardized questionnaire. During this time physical examination was conducted with data collection on blood pressure according to World Health Organization (WHO) guidelines [23] using a semiautomatic digital blood pressure monitor (Rossmax PA, USA) on the right arm in sitting position and anthropometric measurements. Body weight was measured to the nearest 0.1 kg with a Sunbeam EB710 digital bathroom scale, which was calibrated and standardized using a weight of known mass. Measurements were recorded with each subject wearing light clothing, without shoes and socks. Waist circumference was determined using a nonelastic tape at the level of the narrowest part of the torso, as seen from the anterior view. All anthropometric measurements were performed three times and their average was used for analysis. Participants with no history of doctor diagnosed diabetes mellitus underwent a 75 g oral glucose tolerance test (OGTT) as recommended by WHO [24].

### 2.4. Biochemical Analysis

Blood samples were collected from participants after overnight fast and processed as described in [20]. Plasma glucose levels and glycated haemoglobin (HbA1c) in whole blood collected in an EDTA tube were measured, respectively, by enzymatic hexokinase method and the turbidimetric inhibition immunoassay (Cobas 6000, Roche Diagnostics, Germany). Serum insulin was determined by a microparticle enzyme immunoassay (AxSYM, Abbott). High-density lipoprotein cholesterol (HDL-C) and triglycerides (TG) were estimated by enzymatic colorimetric methods in serum (Cobas 6000, Roche Diagnostics). Low-density lipoprotein cholesterol (LDL-C) was calculated using Friedwald's formula [25]. Body mass index (BMI) was calculated as weight/height *∗* height (kg/m^2^) and waist-hip-ratio (WHR) as waist/hip circumference. Diabetes was based on a history of doctor diagnosis, fasting blood glucose concentration ≥ 7.0 mmol/L (or 126 mg/dL), and/or 2-hour post-OGTT plasma glucose ≥ 11.1 mmol/L (or 200 mg/dL).

### 2.5. Genotyping and DNA Methylation

Genomic DNA was extracted from whole blood samples collected in EDTA tubes using the salt extraction method and quantified with the NanoDrop ND-1000 instrument (Nanodrop Technologies, Wilmington, USA). Global DNA methylation was measured with the 5mC DNA ELISA kit according to the manufacturer's instructions (Zymo Research Corp., Irvine, CA, USA). Briefly, 100 ng of genomic DNA was allowed to bind to the enzyme linked immunosorbent assay (ELISA) plate, and the methylated fraction of DNA was detected using a 5-methylcytosine monoclonal antibody and quantified by an ELISA-like reaction and measuring the absorbance at 450 nm. The* MTHFR* rs1801133 and* NOS3* rs1799983 polymorphisms were genotyped using high throughput real-time polymerase chain reaction (RT-PCR) on the BioRad Optica (BioRad, USA) platform using Taqman genotyping assay (Applied Biosystems, USA). Conventional polymerase chain reaction followed by direct DNA sequencing was performed for analytical validation of high throughput genotyping.

### 2.6. Statistical Analysis

General characteristics of the study participants are summarized as count and percentage for dichotomous traits and median and 25th–75th percentiles for quantitative traits. Group comparisons used chi square and Kruskal-Wallis tests. Monotonous trends in the distribution of characteristics across quarters of global DNA methylation were assessed using the Cochran-Armitage trend test for proportions and Jonckheere-Terpstra trend test for medians. Robust regressions were then used to assess the effect of various traits on global DNA methylation in models accounting for age, gender, smoking, and glucose tolerance status. SNPs were tested for departure from Hardy-Weinberg Equilibrium (HWE) expectation via a chi square goodness of fit test, and their effect on DNA methylation was investigated in linear regressions models, while always assuming a log-additive genetic model. Results corresponding to *p* values below 5% are described as significant. All analyses used the statistical software R (version 3.2.2 (2015-08-14), R Foundation for statistical computing, Vienna, Austria). SNPs analyses used the packages “*genetic*” and “*SNPassoc*.”

## 3. Results

### 3.1. Global DNA Methylation and Cardiometabolic Profile

**Figure 1:**
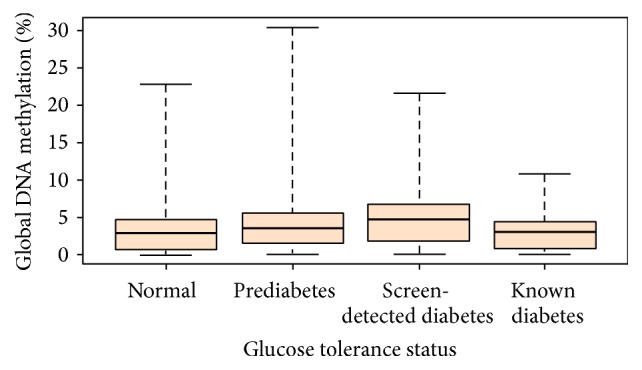
Boxplot showing the distribution of global DNA methylation across glucose tolerance status. Global DNA methylation was significantly higher in diabetic participants than in normotolerant participants (*p* = 0.0003), significantly higher in screen-detected diabetes than in known diabetes (*p* = 0.0188), and significantly higher in prediabetes than in normal glucose tolerance (*p* = 0.0328). But there was no difference in global DNA methylation between known diabetes and normal glucose tolerance (*p* > 0.999) and between known diabetes and prediabetes (*p* = 0.408); all *p* values are adjusted for multiple comparisons. Median global DNA methylation (25th–75th percentiles): normal: 2.81 (0.68–4.62; *N* = 284); prediabetes: 3.40 (1.53–5.45; *N* = 120); screen-detected diabetes: 4.62 (1.71–6.77; *N* = 97); known diabetes: 3.07 (0.80–4.45; *N* = 61).

The study cohort consisted of 564 participants, including 438 (78%) females. One hundred and fifty-eight (28%) individuals had T2DM of which 97 (17.2%) were screen-detected cases. Another one hundred and nineteen (21.1%) had prediabetes, that is, impaired fasting glucose, impaired glucose tolerance, or the combination of both, and the remainder 287 (50.9%) had normal glucose tolerance. Global DNA methylation (%) was significantly higher in subjects with prediabetes or diabetes when compared to individuals with normoglycemia (*p* < 0.05). However, no significant differences were observed between subjects with prediabetes and diabetes. On further analysis, higher global DNA methylation in diabetics was driven by those with screen-detected diabetes. As shown in Figure 1, global DNA methylation was significantly higher in subjects with screen-detected diabetes than in those with normoglycemia (*p* = 0.0003) and remained significantly higher when compared to individuals with known diabetes (*p* = 0.019). However, there was no significant difference between known diabetic and normoglycemia subjects (*p* > 0.999). The characteristics of participants by glucose tolerance status are summarized in Table 1, mostly showing the expected differences.

The baseline characteristics of participants across quarters of global DNA methylation are summarized in Table 2. The proportion of participants with any diabetes increased across increasing quarters of global DNA methylation (*p* = 0.028) and in a linear fashion (*p* = 0.008 for linear trend). Across quarters of global DNA methylation levels, significant differences were also observed in the distribution of fasting (*p* = 0.006) and 2-hour glucose levels (*p* = 0.002), with increasing trends across quarters of global DNA methylation, always in linear fashions (both *p* ≤ 0.003). Significant correlations were also observed between global DNA methylation and diabetes (*r* = 0.101; *p* = 0.016), body mass index and waist circumference (both, *r* = 0.09; *p* = 0.033), fasting blood glucose (*r* = 0.126; *p* = 0.029), and after 2-hour blood glucose (*r* = 0.167; *p* = 0.0002). In robust linear regression analysis adjusted for age, gender, status for hyperglycemia, and smoking, any diabetes (*β* = 0.621; *p* = 0.036) was associated with global DNA methylation. When participants with diabetes were distinguished into those with screen-detected diabetes or known diabetes, the association remained significant only for screen-detected diabetes (*β* = 1.069; *p* = 0.004) but not for known diabetes. In similar regression analyses, no other characteristics emerged as a significant predictor of DNA methylation, although borderline associations were observed with female gender, gamma-GT, and systolic blood pressure (Table 2). The association of any diabetes with DNA methylation also remained significant after expansion of the basic models to include the following correlates of diabetes status in the sample: BMI, waist circumference, systolic blood pressure, drinking status, HDL-cholesterol, total cholesterol, triglycerides, log-transformed CRP, and log-transformed gamma-GT (*β* = 0.653; *p* = 0.038).

### 3.2. Genetic Polymorphisms and DNA Methylation

Table 3 shows the genotype distribution of polymorphisms across the quarters of global DNA methylation. None of the single nucleotide polymorphisms (SNPs) deviated from Hardy-Weinberg Equilibrium (HWE) (all *p* ≥ 0.582), and their distribution showed neither significant difference (all *p* ≥ 0.173) nor linear trends (all *p* ≥ 0.205 for linear trend) across the quarters of global DNA methylation. In linear regression analysis adjusted for age, gender, status for hyperglycemia, and smoking, only T allele of* NOS3* was associated with increasing global DNA methylation with a beta coefficient of 0.943 (95% confidence interval: 0.286 to 1.560) assuming a log-additive genetic model. This association remained significant in sensitivity analysis after exclusion of participants with known diabetes. In this subgroup, the beta coefficient was 1.102 (95% CI: 0.401 to 1.802) for the effect of T allele of* NOS3* on global DNA methylation.

## 4. Discussion

In this study, we measured global DNA methylation in PBMCs of mixed ancestry individuals with different levels of glucose tolerance, in parallel with genetic screening of polymorphisms in the* MTHFR* and* NOS3* genes. Our results show that global DNA methylation is increased in both prediabetic and diabetic states, but this increase was more pronounced in those with screen-detected diabetes, even after adjustment for extraneous factors such as age, gender, smoking, and glucose tolerance status. The distribution of the investigated SNPs did not differ across quarters of DNA methylation, but regression analysis under log-additive genetic model assumption revealed* NOS3* to be an independent determinant of global DNA methylation.

The strengths of our study include a well-characterized cohort and using the reference standard OGTT and a detailed drug history to carefully assign individuals to glucose tolerance status. Both T2DM and DNA methylation are affected by age, obesity, and genetic factors. In this regard, we also evaluated the effect of genes involved in DNA methylation and/or folate metabolism. We quantified DNA methylation across the whole genome using enzyme linked immunosorbent assay (ELISA), which is cost-effective and easy technique that is amenable for routine analysis in developing countries. The use of robust analytic methods also increases the reliability of our findings. For instance, by implementing robust correlations to eliminate the effect of outliers, we provide better estimates of the measures of association without loss of power [26].

In this study, we show that DNA methylation is significantly increased in subjects with screen-detected diabetes compared to those with diabetes and those on treatment. We believe that the lack of association between known diabetes and global DNA methylation in our sample is likely due to the reversal of DNA methylation changes by glucose controls agents in these individuals. These findings are in contrast with a study that quantified global DNA methylation by bisulfite pyrosequencing and showed a null effect of diabetes medication on the association between DNA methylation and insulin resistance [27]. The difference between Zhao et al. [27] and our study may be attributed to the sample size of these two studies. Their study analyzed global DNA methylation of 17 diabetics on glucose control agents, whereas 61 diabetics on medication were included in our study. Genetic polymorphisms have been shown to be responsible for individual responses to many oral antidiabetic drugs [28, 29], and these polymorphisms may also be subject to epigenetic regulation. It is thought that antidiabetic drugs could influence methylation trends through gene expression mechanisms and may represent a possible confounding factor for the identification of T2DM related epigenetic profiles [3]. Furthermore, our results are in contrast with those reported in liver biopsies of subjects with T2DM where genome-wide analysis of DNA methylation identified 94% hypomethylated CpG sites in these individuals [10]. Although diabetic subjects in this study were similarly obese as in Nilsson et al. [10], the differences in the cell types and liver versus blood leucocytes as well as the different methods for quantifying DNA methylation may explain differences in DNA methylation.

Although global DNA methylation correlated positively with adiposity indices, there was no correlation with age. The lack of association between DNA methylation and age is at variance with a number of studies that have repeatedly shown decreasing DNA methylation with increasing age [30–32]. The narrow age range in our study has likely masked any association between DNA methylation and age. We also did not find an association between the MTHFR C677T polymorphism and global DNA methylation, despite previous reports that have shown an association between MTHFR C677T, methyl group generation, and subsequent DNA methylation [33, 34]. Contrary to our prior hypothesis that* MTHFR* gene polymorphisms will affect global DNA methylation, we observed that global DNA methylation was associated with* NOS3* G894T polymorphism. Reduced production of NO is one of the most important contributors to endothelial dysfunction, an initiator of vascular complications [15–19]. NOS3 is encoded by the* NOS3* gene located on chromosome 7q35-36 and three polymorphisms (G894T, 4b/a, and T786C) in the* NOS3* gene are associated with diabetes and diabetes related traits and reduced enzyme activity and consequently bioavailability of NO and endothelial function [19, 35, 36]. It is not clear how NOS3 may influence global DNA methylation, but a study involving obese children and type 1 diabetes with* NOS3* polymorphisms demonstrated an improved endothelial function after supplementation with folate [37]. Similarly, the authors obtained differing results with respect to MTHFR as no improvement in endothelial function was evident in those with* MTHFR* polymorphisms suggesting two independent mechanisms for vascular response to folate for both NOS3 and MTHFR [37]. Folate plays an important role in DNA methylation and several studies have shown aberrant DNA methylation profiles in relation to folate levels [10, 38]. Individuals with T2DM are at an increased risk of both micro- and macrovascular complications and it has previously been shown that glycemic control is not adequate to halt the progression of vascular complications [39–41]. Thus, the observed association between* NOS3* G894T gene polymorphisms and global DNA methylation suggests gene-epigenetic mechanisms through which vascular complications develop despite adequate glycemic control in individuals with T2DM.

The current study represents a significant contribution in the African setting, where research about the contribution of epigenetics to the growing burden of noncommunicable disease in the region is lacking [42]. In summary, our data indicates that global DNA methylation is significantly associated with screen-detected diabetes compared to known diabetes on treatment, leading us to speculate that glucose control agents to some extent may be reversing DNA methylation. Furthermore, we showed that DNA methylation is independent of* MTHFR* polymorphism but is influenced by* NOS3* gene polymorphism G894T. It is known that epigenetic variations are regulated in a tissue-specific manner; however, the use of peripheral blood to assess DNA methylation is recommended for epidemiologic samples [43]. Furthermore, DNA methylation in blood presents a feasible marker for the diagnosis, prevention, and management of T2DM. To fully elucidate the role of DNA methylation in this population, future studies should explore gene-specific methylation together with global DNA methylation analysis.

## Figures and Tables

**Table 1:** Characteristics according to glucose tolerance status.

Characteristic	Normotolerant	Prediabetes	Screen-detected diabetes	Known diabetes	*p* value
*n* (%)	283 (50.5)	119 (21.2)	97 (17.3)	61 (10.9)	
Median actual% 5mc	2.83 (0.71–4.62)	3.41 (1.55–5.45)	4.62 (1.71–6.77)	3.07 (0.80–4.45)	0.0001
Women, *n* (%)	215 (76.0)	98 (82.3)	81 (83.5)	40 (65.6)	0.030
Current smoking, *n* (%)	121 (42.8)	44 (37.0)	35 (36.1)	20 (32.8)	0.363
Current drinking, *n* (%)	80 (28.3)	33 (27.7)	25 (25.8)	12 (19.7)	0.573
Age (years)	49.7 (13.5)	54.0 (12.0)	58.0 (13.3)	58.3 (11.4)	<0.0001
Body mass index (kg/m^2^)	28.7 (7.2)	31.2 (7.1)	32.5 (7.3)	30.5 (6.6)	<0.0001
Waist circumference (cm)	93 (14)	99 (15)	102 (16)	101 (14)	<0.0001
Hip circumference (cm)	108 (15)	112 (14)	114 (15)	109 (15)	0.007
Waist-to-hip ratio	0.86 (0.07)	0.89 (0.09)	0.89 (0.08)	0.92 (0.08)	<0.0001
Systolic blood pressure (mmHg)	119 (18)	122 (15)	129 (21)	128 (18)	<0.0001
Diastolic blood pressure (mmHg)	74 (11)	74 (10)	76 (12)	75 (12)	0.323
Fasting blood glucose (mmol/L)	5.0 (0.7)	5.7 (0.5)	8.5 (3.4)	11.1 (4.2)	<0.0001
2-hour glucose (mmol/L)	5.9 (1.0)	8.6 (1.1)	13.9 (5.7)	NA	<0.0001
HbA1c (%)	5.7 (0.4)	5.8 (0.4)	7.1 (1.8)	8.3 (1.8)	<0.0001
Fasting insulin (*μ*U/mL)	6.2 (3.0–10.6)	7.2 (1.9–12.7)	9.3 (3.7–16.1)	8.0 (2.8–12.8)	0.013
2-hour insulin (*μ*U/mL)	33.9 (17.1–54.4)	58.2 (26.1–97.9)	56.6 (21.2–108.6)	NA	<0.0001
C-reactive protein (mg/L)	2.8 (0.7–7.1)	6.6 (1.8–12.6)	6.4 (2.1–11.8)	4.0 (1.3–7.6)	<0.0001
Cotinine (ng/mL)	10.0 (9.0–307.5)	10.0 (9.0–270.2)	10.0 (9.0–213.0)	10.0 (9.0–125.2)	0.514
Gamma-glutamyltransferase (IU/L)	25.0 (17.0–36.5)	29.0 (22.0–41.0)	33.0 (24.0–54.0)	29.5 (20.0–45.7)	<0.0001
Triglycerides (mmol/L)	1.1 (0.8–1.4)	1.4 (1.1–1.8)	1.6 (1.2–2.1)	1.6 (1.2–2.2)	<0.0001
HDL-cholesterol (mmol/L)	1.3 (0.3)	1.3 (0.3)	1.3 (0.3)	1.1 (0.3)	0.007
LDL-cholesterol (mmol/L)	3.6 (1.0)	3.6 (1.0)	4.0 (1.1)	3.3 (1.1)	0.0001
Total cholesterol (mmol/L)	5.5 (1.2)	5.6 (1.1)	6.1 (1.3)	5.3 (1.1)	<0.0001

Values are count (percentages), mean (standard deviation), or median (25th–75th percentiles).

**Table 2:** Baseline characteristics across quarters of global DNA methylation.

Characteristic	Global DNA methylation				*p* difference^*∗*^	*p*-trend	*r* (*p* value)^*∗∗*^	*β* (*p* value)^*∗∗∗*^
	Q1	Q2	Q3	Q4				
*n*	141	141	140	142				
Actual% 5mc								
Median	0.14	2.14	3.97	7.27				
Women, *n* (%)	100 (70.9)	109 (77.3)	115 (82.1)	114 (80.3)	0.117	0.036	0.076 (0.070)	0.543 (0.063)
Any diabetes	31 (22.0)	38 (26.9)	36 (25.7)	53 (37.3)	0.028	0.008	0.101 (0.016)	0.621 (0.036)
Diabetes status, *n* (%)					0.0007	0.145	0.082 (0.052)	
None	110 (78.0)	103 (73.0)	104 (74.3)	89 (62.7)				*Reference*
Screen-detected	15 (10.6)	23 (16.3)	17 (12.1)	42 (29.6)				1.069 (0.004)
Known diabetes	16 (11.3)	15 (10.6)	19 (13.6)	11 (7.7)				−0.026 (0.947)
Current smoking, *n* (%)	55 (39.0)	60 (42.5)	59 (42.1)	50 (35.2)	0.562	0.519	−0.038 (0.367)	−0.162 (0.505)
Current drinking, *n* (%)	40 (28.4)	37 (26.2)	37 (26.4)	36 (25.3)	0.950	0.594	−0.019 (0.648)	0.211 (0.494)
Age (years)	51 (41–64)	52 (44–60)	53 (44–62)	53 (44–64)	0.883	0.523	0.036 (0.391)	0.007 (0.443)
Body mass index (kg/m^2^)	29.1 (24.4–33.4)	28.8 (23.6–34.7)	28.6 (24.3–34.3)	31.0 (26.3–34.9)	0.209	0.051	0.090 (0.033)	0.015 (0.464)
Waist circumference (cm)	96.0 (85.5–104.0)	96.5 (85.1–108.0)	97.0 (87.5–106.4)	99.5 (88.4–108.2)	0.256	0.053	0.090 (0.033)	0.007 (0.444)
Hip circumference (cm)	107.8 (98.0–116.5)	108.0 (99.2–118.0)	109.2 (100.2–117.5)	110.0 (101.5–121.0)	0.385	0.083	0.083 (0.050)	0.004 (0.685)
Waist-to-hip ratio	0.87 (0.82–0.93)	0.87 (0.83–0.93)	0.87 (0.84–0.92)	0.88 (0.83–0.93)	0.871	0.477	0.031 (0.465)	1.265 (0.470)
Systolic blood pressure (mmHg)	121 (109–137)	120 (109–134)	119 (108–129)	120 (109–133)	0.570	0.514	−0.025 (0.557)	−0.012 (0.090)
Diastolic blood pressure (mmHg)	75 (66–82)	74 (67–83)	73 (66–79)	73 (67–82)	0.529	0.379	−0.039 (0.357)	−0.013 (0.207)
Fasting blood glucose (mmol/L)	5.4 (4.9–6.0)	5.7 (5.0–6.3)	5.4 (5.0–6.3)	6.0 (5.0–7.1)	0.006	0.003	0.126 (0.029)	0.066 (0.225)
2-hour glucose (mmol/L)	6.6 (5.4–9.0)	6.9 (6.0–8.8)	7.0 (5.6–8.6)	7.8 (6.0–10.0)	0.002	0.0003	0.167 (0.0002)	0.011 (0.824)
HbA1c (%)	5.7 (5.5–6.1)	5.8 (5.5–6.3)	5.9 (5.6–6.3)	5.9 (5.5–6.3)	0.519	0.195	0.057 (0.181)	−0.039 (0.782)
Fasting insulin (*μ*U/mL)	7.3 (3.7–11.7)	6.1 (2.1–12.9)	6.7 (2.9–12.4)	8.3 (3.0–12.8)	0.435	0.961	−0.005 (0.900)	−0.016 (0.133)
2-hour insulin (*μ*U/mL)	36.3 (19.9–64.3)	38.7 (18.3–68.4)	39.7 (10.2–68.1)	44.1 (19.5–89.4)	0.485	0.131	0.070 (0.120)	0.0004 (0.860)
C-reactive protein (mg/L)	3.6 (0.9–9.2)	4.4 (1.1–9.6)	2.8 (0.9–9.4)	5.3 (1.7–11.7)	0.091	0.121	0.080 (0.059)	0.005 (0.641)
Cotinine (ng/mL)	10.0 (9.0–282.1)	10 (9–310)	10 (9–270)	10.0 (9.0–197.2)	0.417	0.323	−0.059 (0.160)	−0.002 (0.109)
Gamma-glutamyltransferase (IU/L)	27.0 (18.0–40.0)	28.0 (20.0–45.7)	27.5 (19.0–39.2)	28.0 (20.2–44.5)	0.820	0.625	0.0005 (0.991)	−0.002 (0.081)
Triglycerides (mmol/L)	1.23 (0.93–1.74)	1.24 (0.90–1.69)	1.27 (0.87–1.81)	1.34 (1.06–1.92)	0.096	0.048	0.079 (0.061)	−0.065 (0.642)
HDL-cholesterol (mmol/L)	1.28 (1.11–1.46)	1.22 (0.99–1.45)	1.24 (1.03–1.45)	1.16 (0.99–1.44)	0.138	0.058	−0.081 (0.055)	−0.509 (0.130)
LDL-cholesterol (mmol/L)	3.49 (2.84–4.28)	3.43 (2.80–4.05)	3.62 (3.00–4.26)	3.68 (2.90–4.39)	0.279	0.136	0.079 (0.062)	0.078 (0.538)
Total cholesterol (mmol/L)	5.52 (4.70–6.40)	5.33 (4.59–6.05)	5.59 (4.75–6.37)	5.54 (4.93–6.46)	0.183	0.275	0.061 (0.147)	−0.009 (0.933)

^*∗*^ *p* values from Kruskal-Wallis and chi square tests for the differences across quarters of global DNA methylation.

^*∗∗*^Spearman correlation coefficients and *p* values for the continuous associations of global DNA methylation with covariates.

^*∗∗∗*^Beta coefficients and *p* values from age, gender, and status for hyperglycemia and smoking adjusted robust linear regressions for the prediction of global DNA methylation by various traits.

**Table 3:** Genotype distribution across the quarters of global DNA methylation.

Characteristic	Global DNA methylation				*p* difference^*∗*^	*p*-trend	*p* HWE	*β* (95% CI)^*∗∗∗*^
	Q1	Q2	Q3	Q4				
*n*	111	110	112	111				
Actual% 5mc								
Median	0.11	2.12	3.94	7.24				
MTHFR rs1801133					0.665	0.255	0.697	−0.387 (−1.054 to 0.281)
C/C	75 (68.8)	78 (71.6)	85 (76.6)	86 (77.5)				
C/T	32 (29.4)	27 (24.8)	24 (21.6)	23 (20.7)				
T/T	2 (1.8)	4 (3.7)	2 (1.8)	2 (1.8)				
NOS3 rs1799983					0.173	0.205	0.582	0.943 (0.286 to 1.560)
G/G	82 (76.6)	73 (68.9)	81 (77.1)	65 (62.5)				
G/T	23 (21.5)	28 (26.4)	22 (20.9)	36 (34.6)				
T/T	2 (1.9)	5 (4.7)	2 (1.9)	3 (2.9)				

^*∗*^ *p* values from Kruskal-Wallis and chi square tests for the differences across quarters of global DNA methylation.

^*∗∗∗*^Beta coefficients and 95% confidence interval from age, gender, and status for hyperglycemia and smoking adjusted linear regressions for the prediction of global DNA methylation by various SNPs assuming a log-additive genetic model.

HWE: Hardy-Weinberg equilibrium.
